# Estimating Progression-Free Survival in Patients with Primary High-Grade Glioma Using Machine Learning †

**DOI:** 10.3390/jcm13206172

**Published:** 2024-10-16

**Authors:** Agnieszka Kwiatkowska-Miernik, Piotr Gustaw Wasilewski, Bartosz Mruk, Katarzyna Sklinda, Maciej Bujko, Jerzy Walecki

**Affiliations:** 1 Centre of Radiological Diagnostics, National Medical Institute of the Ministry of the Interior and Administration, Wołoska 137, 02-507 Warsaw, Poland; 2 Department of Neurosurgery, National Medical Institute of the Ministry of the Interior and Administration, Wołoska 137, 02-507 Warsaw, Poland

**Keywords:** radiomics, glioma, artificial intelligence, recurrence

## Abstract

**Background/Objectives**: High-grade gliomas are the most common primary malignant brain tumors in adults. These neoplasms remain predominantly incurable due to the genetic diversity within each tumor, leading to varied responses to specific drug therapies. With the advent of new targeted and immune therapies, which have demonstrated promising outcomes in clinical trials, there is a growing need for image-based techniques to enable early prediction of treatment response. This study aimed to evaluate the potential of radiomics and artificial intelligence implementation in predicting progression-free survival (PFS) in patients with highest-grade glioma (CNS WHO 4) undergoing a standard treatment plan. **Methods**: In this retrospective study, prediction models were developed in a cohort of 51 patients with pathologically confirmed highest-grade glioma (CNS WHO 4) from the authors’ institution and the repository of the Cancer Imaging Archive (TCIA). Only patients with confirmed recurrence after complete tumor resection with adjuvant radiotherapy and chemotherapy with temozolomide were included. For each patient, 109 radiomic features of the tumor were obtained from a preoperative magnetic resonance imaging (MRI) examination. Four clinical features were added manually—sex, weight, age at the time of diagnosis, and the lobe of the brain where the tumor was located. The data label was the time to recurrence, which was determined based on follow-up MRI scans. Artificial intelligence algorithms were built to predict PFS in the training set (n = 75%) and then validate it in the test set (n = 25%). The performance of each model in both the training and test datasets was assessed using mean absolute percentage error (MAPE). **Results**: In the test set, the random forest model showed the highest predictive performance with 1-MAPE = 92.27% and a C-index of 0.9544. The decision tree, gradient booster, and artificial neural network models showed slightly lower effectiveness with 1-MAPE of 88.31%, 80.21%, and 91.29%, respectively. **Conclusions**: Four of the six models built gave satisfactory results. These results show that artificial intelligence models combined with radiomic features could be useful for predicting the progression-free survival of high-grade glioma patients. This could be beneficial for risk stratification of patients, enhancing the potential for personalized treatment plans and improving overall survival. Further investigation is necessary with an expanded sample size and external multicenter validation.

## 1. Introduction

Glioma is a brain tumor associated with a high mortality rate [1]. According to the World Health Organization’s (WHO) classification of central nervous system (CNS) tumors, grade 4 tumors are the most common primary malignant brain tumors in adults [2]. These neoplasms remain predominantly incurable due to, among others, the genetic diversity within each tumor, leading to varied responses to specific drug therapies [3]. Currently, the most widely used therapy around the world for primary high-grade gliomas is the so-called new Stupp protocol, which consists of maximal surgical resection of the tumor with adjuvant radiotherapy (RT) and chemotherapy (CHTH) with temozolomide (TMZ) [4,5,6]. The implementation of this therapy is associated with an extension of the mean overall survival to 14.6 months [7]. However, with the emergence of new targeted and immune therapies, which have demonstrated promising results in clinical trials, there is a growing need for image-based methods to predict treatment response.

**Figure 1:**
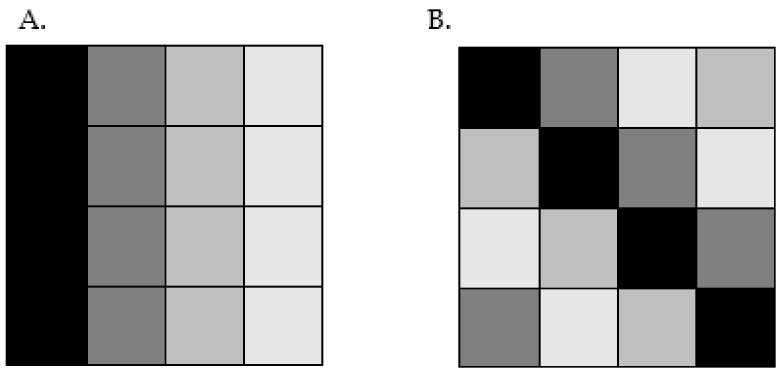
Assuming that each small square represents a pixel, the morphological and first-order features of images (**A**,**B**) would be the same, but the images differ in texture.

The answer to this growing demand may lie in the application of artificial intelligence, which is increasingly being used in medicine, especially in radiology. One of the subfields of radiology today is radiomics, which is characterized by the extraction of new data in quantitative form from radiological images. Analysis of such acquired data by artificial intelligence models leads to the discovery of new clinically important information in radiological images. In daily clinical practice, visual analysis of images is based on qualitative descriptors (such as signal intensity, density, heterogeneity, and level of contrast enhancement) or simple quantitative characteristics (for example, dimension, volume, and number of lesions). The sensitivity of such measurements is not high and strongly dependent on the experience of the evaluating radiologist. By automatically extracting the same features from imaging data, computational methods eliminate these disadvantages. These new digital features, which are not intuitively recognizable by humans, are called radiomic features and can be divided into three main groups:

1. Morphological features, which particularly describe the size and shape of the previously segmented region of interest.
2. First-order features, which are based on a histogram of pixel/voxel intensities of the region of interest.
3. Second-order features, which describe the texture of the region of interest. Texture-based features are the most complex and describe the heterogeneity of the image. In radiomics, they are also often the most important due to the more accurate description of the image. Figure 1 shows an example of two images that, when described by radiomic features, would not differ in morphological features and first-order features but differ significantly in texture.

Neuro-oncology is one of the specialties in which the advances in radiomics are the most noticeable. Numerous studies have evaluated the use of radiomics models in predicting the presence of mutations in gliomas or the time to recurrence of gliomas after treatment [8]. Ailing He et al. proposed a radiomics model based on MRI images to predict IDH mutation status in low-grade gliomas. The model performed well in validation datasets with an AUC of 0.873 [9]. Jiangwei Lao et al. proposed a radiomics model for the prediction of overall survival in glioblastoma multiforme. The model performed OS prediction with a C-index  =  0.710 [10].

However, to the best of the authors’ knowledge, there is currently no adequate clinical or image-based predictive model to predict precise PFS (calculated in days) in patients with the highest-grade glioma treated uniformly. Therefore, this study aimed to develop and evaluate an AI model based on radiomic features for prediction of progression-free survival (PFS) in patients with the highest-grade glioma (CNS WHO 4) undergoing a standard treatment plan. The developed machine learning model can help clinicians identify patients who are most likely to benefit from the standard treatment plan and support the implementation of personalized therapy.

## 2. Materials and Methods

This study carried out a self-evaluation using METRICS, the details of which are provided in Supplementary S1 [11].

**Figure 2:**
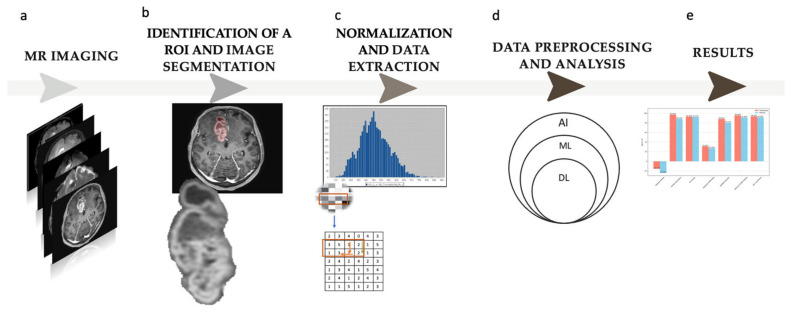
Study flowchart. (**a**) Magnetic resonance (MR) imaging; the study is based on contrast-enhanced T1—w images. (**b**) Identification of a region of interest (ROI) and semi-automatic image segmentation. (**c**) Normalization and radiomic feature extraction from the defined ROI; 109 radiomic features were obtained in the study. (**d**) Data preprocessing and analysis; five different machine learning (ML) models were trained on the received data (AI—artificial intelligence, DL—deep learning). (**e**) Results.

The flowchart of the study is shown in Figure 2.

### 2.1. Study Group

This retrospective study analyzed the medical records of 210 adult patients with pathologically confirmed primary high-grade glioma (CNS WHO 4). As the study included patients hospitalized before 2021, the diagnosis of glioblastoma multiforme fulfilling the World Health Organization’s criteria at that time now corresponds to both glioblastoma CNS WHO 4 and astrocytoma CNS WHO 4 [2]. The inclusion criteria were as follows:

- A histopathologic diagnosis of glioma CNS WHO 4;
- Available preoperative MR imaging, including contrast-enhanced T1WI;
- Available follow-up MR imaging with a reported recurrence or imaging follow-up up to two years that showed no features of tumor recurrence.

The exclusion criteria were as follows:

- Artifacts in MR images;
- Treatment program other than complete resection with adjuvant treatment consisting of temozolomide chemotherapy and radiotherapy;
- Inconclusive result of the follow-up MR examination.

A total of 51 patients were ultimately included in the study.

Data were obtained from the authors’ institution and the publicly available Cancer Genome Atlas Glioblastoma (TCGA-GBM) clinical database and the associated imaging data from the Cancer Imaging Archive (TCIA) [12,13].

**Figure 3:**
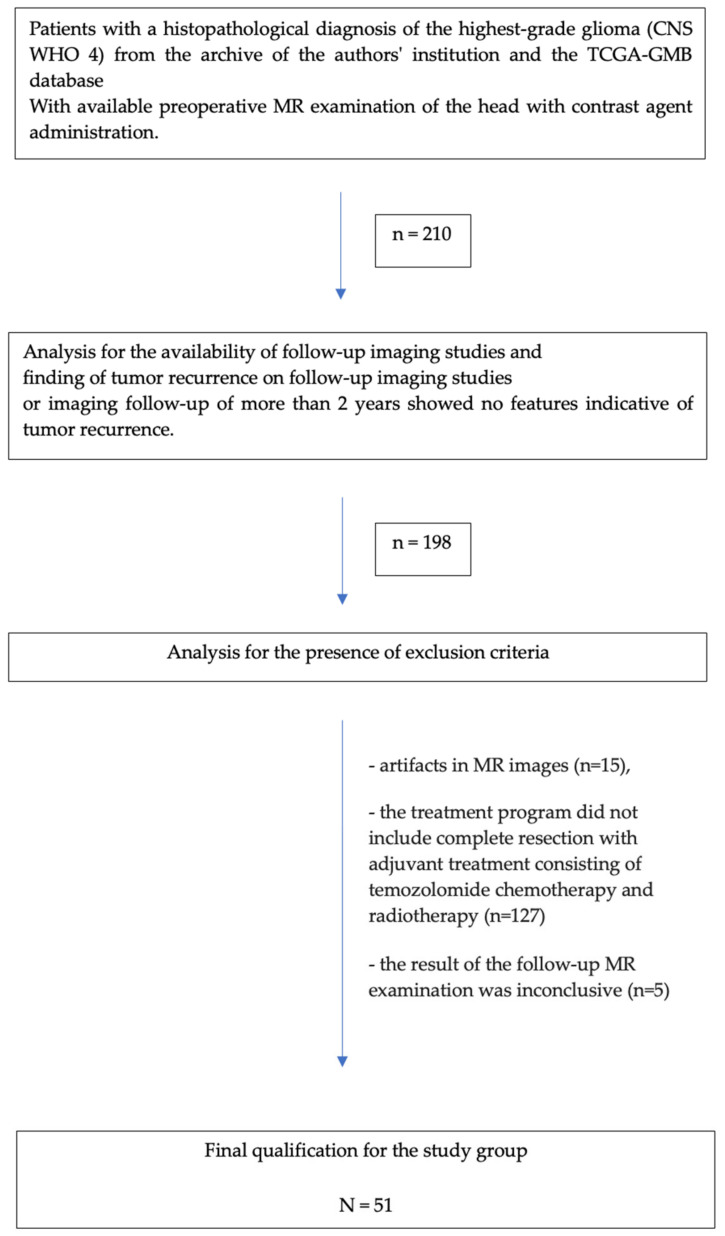
Flowchart of the patient selection process.

The detailed patient selection process is shown in Figure 3. The main exclusion factor was the standard treatment plan, as some patients from the TCGA-GBM database received bevacizumab, cisplatin, or targeted molecular therapy, among others, as adjuvant chemotherapy instead of temozolomide.

### 2.2. Identification of a Region of Interest (ROI) and Segmentation

**Figure 4:**
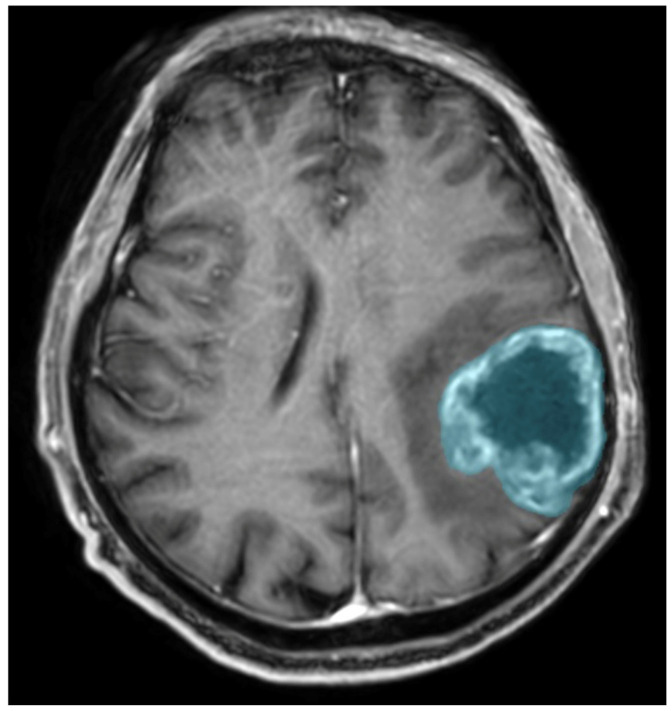
Glioma CNS WHO 4 in the left parietal lobe. T1-weighted image after administration of contrast agent; the blue color was used to mark the tumor segmented by the semi-automated method.

Preoperative MRIs were analyzed using Syngo.Via VB10, Research Frontier, Siemens Healthineers. Segmentation of the lesion was performed semi-automatically by a single reader (a fourth-year radiology resident). The 2D region of interest (ROI) was marked on the axial view of contrast-enhanced T1-weighted imaging (CE T1WI) on the cross-section in which the tumor had the largest area, and the zone of visible contrast enhancement was identified as the tumor boundary. An example of semi-automated tumor segmentation is shown in Figure 4.

### 2.3. Normalization and Extraction of the Data

Given that the database included data from multiple sites, there were variations in scanner models, pixel spacing, slice thickness, and contrast within the selected cohort. Normalization was carried out to account for these differences using Syngo.Via VB10, Research Frontier, Siemens Healthineers. All images were resampled to a common voxel resolution of 1 mm^3^, and the intensities within each voxel were normalized to a [0, 1] range.

For each patient, 109 radiomic features of the tumor were obtained, and feature groups used in the study are shown in Table 1. Four features were added manually—sex, weight, age at the time of diagnosis, and the lobe of the brain where the tumor was located. The data label was the time to recurrence (calculated in days), which was determined based on follow-up MRI exams evaluated by an experienced radiologist.

### 2.4. Data Preprocessing

Reducing the number of features was essential, as the large set of 109 radiomic features could lead to overfitting when predicting progression-free survival (PFS). Additionally, some features might have no variance, be highly correlated with others, or be minimally relevant to PFS prediction. The authors employed methods like locally linear embedding (LLE) and principal component analysis (PCA) to enhance the model’s generalizability and obtain higher model performance. Tools for data multiplication of the learning set were used by generating non-repeating data from existing examples while fully considering the underlying patterns and relationships. With the artificial neural network algorithm, a data compression tool based on an encoder–decoder neural network architecture was used.

The dataset was randomly split into training and validation sets in a 75:25 ratio. The training set was utilized to build the predictive model, while the validation set was used for an independent assessment of the model’s performance. The details of data preprocessing are shown in Table 2.

Among the clinical data, confounding factors were analyzed. None of the added clinical data (gender, age, and weight) individually had a statistically significant effect on PFS (*p* < 0.01); therefore, they were not excluded.

### 2.5. Development and Validation of Models

Progression-free survival (PFS) was defined as the time from therapy initiation to identification of tumor recurrence on MRI follow-up examination. If the patient was not found to have a tumor recurrence at the last follow-up, the PFS was censored at the time of the last follow-up (at least 2 years).

Due to the diverse properties of artificial intelligence models, we utilized five different machine learning models: decision tree (DT), random forest (RF), support vector machine (SVM), gradient boosting (GBoost), and artificial neural network (ANN).

For statistical analyses, Python Version 3.12 was used. Mean absolute percentage error (MAPE) was used to assess the performance of the models.

## 3. Results

### 3.1. Patient Characteristics

**Figure 5:**
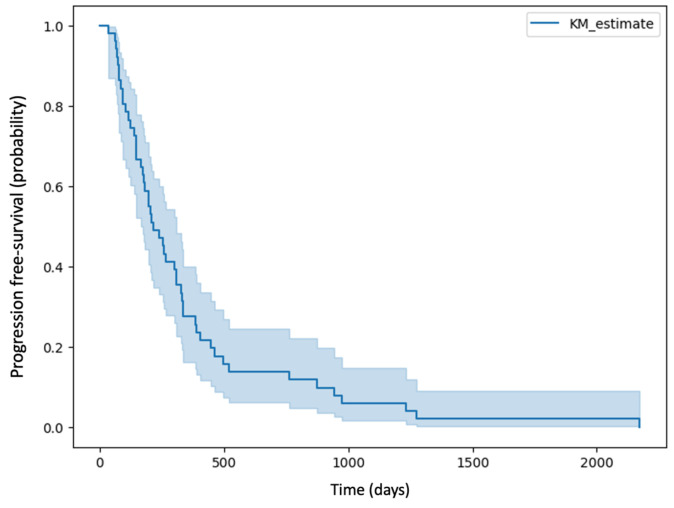
Kaplan–Meier curve of PFS for patients in the study group.

A total of 51 patients were included in the study, including 17 women and 34 men. The mean age of the patients included in the study was 56 years, and the median was 59 years. The mean time to recurrence was 352 days, and the median was 215 days. The Kaplan–Meier curve of PFS for patients in the study group is shown in Figure 5.

### 3.2. Model Interpretation

There are several methods to evaluate the performance of a regression model. A common traditional approach is to measure how accurately the predictions match the actual outcomes [14]. This can be carried out using methods like mean absolute error (MAE), mean squared error (MSE), root mean squared error (RMSE), R-squared (R^2^), and mean absolute percentage error (MAPE). Another method is the C-index (concordance index). The performance of each model in both the training and test datasets is shown in Table 3 and Table 4.

**Figure 6:**
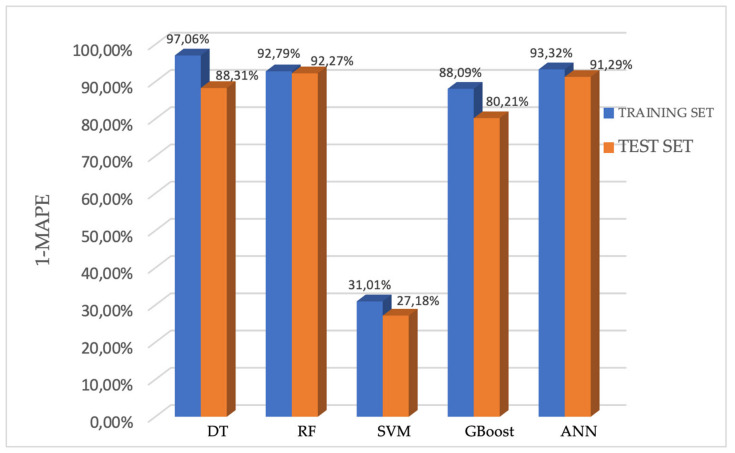
Performance of the five models for predicting the PFS presented using 1-MAPE.

The values of 1-MAPE for each model are shown in Figure 6.

In the training set, the 1-MAPE of the DT, RF, SVM, GBoost, and ANN models were 97.06%, 92.79%, 31.01%, 88.09%, and 93.32%, respectively. Accordingly, in the testing set, the 1-MAPE of these five models were 88.31%, 92.27%, 27.18%, 80.21%, and 91.29%, respectively.

**Figure 7:**
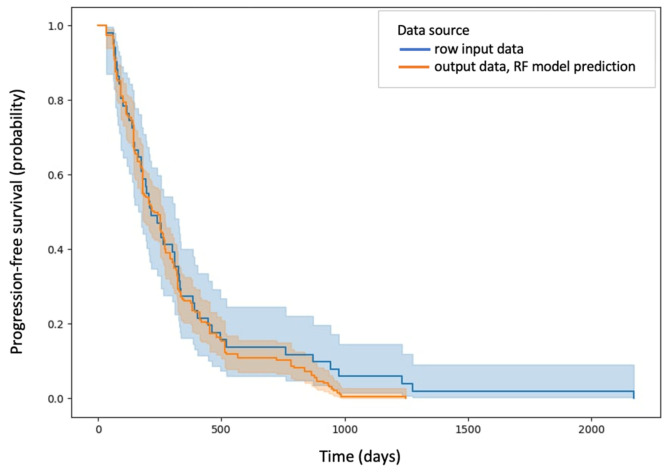
Kaplan–Meier curve of predicted PFS for the test set by the random forest model marked in blue and Kaplan–Meier curve of PFS for patients in the study group marked in orange.

The estimation performance of the DT, RF, GBoost and ANN models reached values above 80% on the test set, which means that these models predict the PFS (calculated in days) with more than 80% accuracy. Among them, the random forest model showed the highest efficiency, predicting PFS on the test set with 92.27% accuracy (C-index: 0.95). The Kaplan–Meier curve of predicted PFS for the test set by the random forest model is shown in Figure 7 together with the Kaplan–Meier curve of PFS for patients in the study group.

As data multiplication and reduction of dimensionality were used, it was not possible to obtain exact information on which radiomic features made the greatest contribution to the results.

## 4. Discussion

The results of our study indicate that based on radiomic analysis, which involves extracting texture and morphologic features from numerous medical images in combination with artificial intelligence models, it is possible to build a group of models with a high predictive ability of progression-free survival (PFS). By utilizing advanced computational techniques, these AI-driven models can process vast amounts of radiomic data and identify subtle patterns and correlations, resulting in high prediction performance.

Medical imaging data are predicted to soon represent 30% of global data storage [15]. It is important to use them as efficiently as possible. Machine learning models used to analyze them appear to be key. Therefore, it is important to use several algorithms to analyze the same radiomic data, primarily to expand our knowledge of it. The results of this study show that some of the algorithms have similar accuracy, and the accuracy of the support vector machine (SVM) model is insufficient. However, in a study conducted by Rachel Zhao et al., in which the authors used machine learning models based on clinical data to predict time to recurrence, the accuracy of the SVM model was the highest among the applied models, and the random forest (RF) model had the lowest accuracy (C-index 0.767, 0.771, and 0.57 for Cox proportional hazard (CPH), SVM, and RF models, respectively) [16]. This supports the appropriateness of using several algorithms to analyze the same data.

The implementation of an artificial intelligence (AI) algorithm able to predict recurrence-free time (PFS) with up to 92% accuracy in patients diagnosed with WHO 4 CNS gliomas creates new possibilities in clinical decision-making of these most aggressive brain tumors. One of the main challenges in treating gliomas is their heterogeneity, their ability to infiltrate surrounding brain structures, and, perhaps related to this, their tendency to recur [3]. These features make glioma CNS WHO 4 extremely difficult to treat. Even with a combination of surgical treatment, radiation therapy, and chemotherapy, most patients experience recurrence within a relatively short time. Therefore, a key aspect of improving treatment outcomes is the ability to predict recurrence early. Accurate diagnosis of PFS before treatment could directly influence the choice of treatment options, enhancing the potential for personalized treatment. In addition, it can be a way to find early so-called long-term survivors (LTSs) and extreme long-term survivors (ELTSs). In the glioma CNS WHO 4 patient population, LTSs represent 13% of patients and are characterized by survival of at least 2 years, while ELTSs represent <1% of patients and their survival time is >10 years [17,18]. Identifying this group as early as possible at the beginning of the diagnostic pathway would be extremely significant for clinicians and patients.

Our study has several limitations that should be noted. Firstly, the retrospective nature of the study and the small sample size limit the generalizability of our findings. Despite efforts to mitigate overfitting caused by high dimensionality through techniques such as dimension reduction and data augmentation, the prediction model still requires further validation with a larger dataset. Future research should focus on multicenter studies with larger sample sizes and prospective designs to confirm the model’s broader applicability.

Secondly, the process of semi-automatic segmentation of the 2D tumor region of interest (ROI) may impact reproducibility and is a labor-intensive task. Adopting an automatic 3D tumor segmentation algorithm based on deep learning could significantly improve reproducibility and streamline the analysis process, making it more feasible for large-scale data applications.

Thirdly, our study did not incorporate multimodal MRI images, which could potentially enhance the performance and accuracy of the predictive model. Including various imaging modalities in future research could provide more comprehensive data and improve model outcomes.

Lastly, the biological relevance of the radiomic features used in our study is not yet fully understood. Further research is needed to elucidate the connections between radiomics data and tumor biology, which could enhance the interpretability and clinical utility of the predictive models.

## 5. Conclusions

The results of our study show that artificial intelligence models combined with radiomic features could be useful for predicting the progression-free survival of high-grade glioma patients. This could be beneficial for risk stratification of patients, enhancing the potential for personalized treatment plans and improving overall survival.

## Figures and Tables

**Table 1:** Groups of radiomic features used in the study.

Feature Group	Number of Features in Each Group
First-order features based on image intensity histogram descriptors	18
Features describing size and shape	16
Features describing the texture extracted from analysis:	75
- Gray-level co-occurrence matrices (GLCM)	24
- Run-length matrix (RLM)	16
- Size-zone matrix (SZM)	16
- Neighboring gray tone difference matrix (NGTDM)	5
- Gray-level run-length matrix (GLRLM)	14

**Table 2:** The details of data preprocessing.

ML Model	Task	Data Multiplication	Dimensionality Reduction
Decision tree (DT)	Classification	×19	Dimensional reduction of up to 11
Random forest (RF)	Classification		
Support vector machine (SVM)	Classification		
Gradient boosting (GBoost)	Classification		
Artificial neural network (ANN)	Classification	×19	Dimensional reduction of up to 66

**Table 3:** Results of each model in the training set presented using mean absolute error, mean squared error, root mean squared error, R^2^ score, mean absolute percentage error, and 1-mean absolute percentage error.

	Decision Tree (DT)	Random Forest (RF)	Support Vector Machine (SVM)	Gradient Boosting (GBoost)	Artificial Neural Network (ANN)
Mean absolute error	7.4973	19.1089	180.0376	21.8983	33.9342
Mean squared error	254.1967	3250.7404	92,385.7830	900.2685	8942.9704
Root mean squared error	15.9435	57.0153	303.9503	30.0045	94.5673
R^2^ score	0.9969	0.9606	−0.1200	0.9891	0.8799
Mean absolute percentage error	2.9382	7.2073	68.9886	11.9109	6.6754
1-mean absolute percentage error	97.0618	92.7927	31.0114	88.0891	93.3246

**Table 4:** Results of each model in the test set presented using mean absolute error, mean squared error, root mean squared error, R^2^ score, mean absolute percentage error, 1-mean absolute percentage error, C-index.

	Decision Tree (DT)	Random Forest (RF)	Support Vector Machine (SVM)	Gradient Boosting (GBoost)	Artificial Neural Network (ANN)
Mean absolute error	24.8663	25.4891	178.8463	37.3006	45.8352
Mean squared error	4931.2071	4005.8066	79,090.9032	3712.5782	11,766.1994
Root mean squared error	70.2226	63.2914	281.2310	60.9309	108.4721
R^2^ score	0.9282	0.9417	−0.1514	0.9460	0.8706
Mean absolute percentage error	11.6877	7.7275	72.8181	19.7899	8.7136
1-mean absolute percentage error	88.3123	92.2725	27.1819	80.2101	91.2964
C-index	0.9413	0.9544	0.5743	0.9254	0.9284

## Data Availability

The results published here are partly based on data generated by the TCGA Research Network: http://cancergenome.nih.gov/ (accessed on 10 January 2024).

## Supplementary Materials

The following supporting information can be downloaded at https://www.mdpi.com/article/10.3390/jcm13206172/s1, Supplementary S1. Self-evaluation using METhodological RadiomICs Score (METRICS).
