# Supplementary material for: Estimating Progression-Free Survival in Patients with Primary High-Grade Glioma Using Machine Learning
Source: J Clin Med. 2024 Oct 16;13(20):6172. doi: 10.3390/jcm13206172 (PMC11508924; doi:10.3390/jcm13206172)
Supplement: Supplementary file 1 [file jcm-13-06172-s001.zip › jcm-3231738-supplementary.pdf]

METRICS Tool v1.0

Please fill out all conditions first for relevant sections and then all active items to calculate METRICS score.

Please note that default option is "No".

? Stands for explanation of items and conditions.

C Stands for conditional items or sections.

Items/Conditions	Definitions	Weights	Options
**Study Design**			
Item#1	? Adherence to radiomics and/or machine learning-specific checklists or guidelines	0.0368	◉ Yes ○ No
Item#2	? Eligibility criteria that describe a representative study population	0.0735	◉ Yes ○ No
Item#3	? High-quality reference standard with a clear definition	0.0919	◉ Yes ○ No
**Imaging Data**			
Item#4	? Multi-center	0.0438	◉ Yes ○ No
Item#5	? Clinical translatability of the imaging data source for radiomics analysis	0.0292	○ Yes ◉ No
Item#6	? Imaging protocol with acquisition parameters	0.0438	○ Yes ◉ No
Item#7	? The interval between imaging used and reference standard	0.0292	◉ Yes ○ No
**Segmentation** C			
Condition#1	? Does the study include segmentation?		◉ Yes ○ No
Condition#2	? Does the study include fully automated segmentation?		○ Yes ◉ No
			◉ Yes

Item#8	?	Transparent description of segmentation methodology	0.0337	No
Item#9	?	Formal evaluation of fully automated segmentation C	0.0225	Yes / No
Item#10	?	Test set segmentation masks produced by a single reader or automated tool	0.0112	Yes (selected) / No

Image Processing and Feature Extraction

Condition#3	?	Does the study include hand-crafted feature extraction?		Yes (selected) / No
Item#11	?	Appropriate use of image preprocessing techniques with transparent description	0.0622	Yes (selected) / No
Item#12	?	Use of standardized feature extraction software C	0.0311	Yes (selected) / No
Item#13	?	Transparent reporting of feature extraction parameters, otherwise providing a default configuration statement	0.0415	Yes / No (selected)

Feature Processing

Condition#4	?	Does the study include tabular data?		Yes (selected) / No
Condition#5	?	Does the study include end-to-end deep learning?		Yes (selected) / No
Item#14	?	Removal of non-robust features C	0.0200	Yes / No (selected)
Item#15	?	Removal of redundant features C	0.0200	Yes / No (selected)
Item#16	?	Appropriateness of dimensionality compared to data size C	0.0300	Yes / No (selected)
Item#17	?	Robustness assessment of end-to-end deep learning pipelines C	0.0200	Yes / No (selected)

Preparation for Modeling

Item#18	?	Proper data partitioning process	0.0599	Yes (selected) / No

Item#19	?	Handling of confounding factors	0.0300	(•) Yes ( ) No

Metrics and Comparison

Item#20	?	Use of appropriate performance evaluation metrics for task	0.0352	(•) Yes ( ) No
Item#21	?	Consideration of uncertainty	0.0234	(•) Yes ( ) No
Item#22	?	Calibration assessment	0.0176	( ) Yes (•) No
Item#23	?	Use of uni-parametric imaging or proof of its inferiority	0.0117	(•) Yes ( ) No
Item#24	?	Comparison with a non-radiomic approach or proof of added clinical value	0.0293	(•) Yes ( ) No
Item#25	?	Comparison with simple or classical statistical models	0.0176	( ) Yes (•) No

Testing

Item#26	?	Internal testing	0.0375	( ) Yes (•) No
Item#27	?	External testing	0.0749	( ) Yes (•) No

Open Science

Item#28	?	Data availability	0.0075	( ) Yes (•) No
Item#29	?	Code availability	0.0075	( ) Yes (•) No
Item#30	?	Model availability	0.0075	( ) Yes (•) No

Total METRICS score: 61.7%

? **Quality category: Good**

? **Publication ID:**

If you publish any work which uses this tool, please cite the following publication:

Kocak B, Akinci D'Antonoli T, Mercaldo N, et al. METhodological RadiomICs Score (METRICS): a quality scoring tool for radiomics research endorsed by EuSoMII. Insights Imaging. 2024;15(1):8. Published 2024 Jan 17. doi:10.1186/s13244-023-01572-w
